# *NSD1*- and *NSD2*-damaging mutations define a subset of laryngeal tumors with favorable prognosis

**DOI:** 10.1038/s41467-017-01877-7

**Published:** 2017-11-24

**Authors:** Suraj Peri, Evgeny Izumchenko, Adrian D. Schubert, Michael J. Slifker, Karen Ruth, Ilya G. Serebriiskii, Theresa Guo, Barbara A. Burtness, Ranee Mehra, Eric A. Ross, David Sidransky, Erica A. Golemis

**Affiliations:** 1 Biostatistics and Bioinformatics Division, Fox Chase Cancer Center, Philadelphia, 19111 PA USA; 2 Department of Otolaryngology-Head and Neck Surgery, Johns Hopkins University School of Medicine, Baltimore, MD 21287 USA; 3 Molecular Therapeutics Program, Fox Chase Cancer Center, Philadelphia, PA 19111 USA; 4 Department of Oncology, Johns Hopkins University School of Medicine, Baltimore, MD 21287 USA; 5 Department of Internal Medicine and Developmental Therapeutics Program, Yale Cancer Center, Yale School of Medicine, Yale University, New Haven, CT 06520 USA; 6 Department of Otorhinolaryngology-Head and Neck Surgery, Inselspital, University Hospital and University of Bern, Bern, 3010 Switzerland

## Abstract

Squamous cell carcinomas of the head and neck (SCCHN) affect anatomical sites including the oral cavity, nasal cavity, pharynx, and larynx. Laryngeal cancers are characterized by high recurrence and poor overall survival, and currently lack robust molecular prognostic biomarkers for treatment stratification. Using an algorithm for integrative clustering that simultaneously assesses gene expression, somatic mutation, copy number variation, and methylation, we for the first time identify laryngeal cancer subtypes with distinct prognostic outcomes, and differing from the non-prognostic laryngeal subclasses reported by The Cancer Genome Atlas (TCGA). Although most common laryngeal gene mutations are found in both subclasses, better prognosis is strongly associated with damaging mutations of the methyltransferases *NSD1* and *NSD2*, with findings confirmed in an independent validation cohort consisting of 63 laryngeal cancer patients. Intriguingly, NSD1/2 mutations are not prognostic for nonlaryngeal SCCHN. These results provide an immediately useful clinical metric for patient stratification and prognostication.

## Abstract (web summary)

The authors use an integrative clustering approach to identify two laryngeal cancer clusters with distinct prognosis and show that mutations damaging the NSD1 and NSD2 methyltransferases segregate to the cluster with favorable prognosis, and independently predict longer survival in patients with laryngeal, but not other head and neck cancers.

## Introduction

Squamous cell carcinomas of the head and neck (SCCHN) are highly heterogeneous, arise from multiple anatomical sites, and are characterized by aggressive local invasion and overall poor prognosis. While oropharyngeal SCCHN is subclassified into human papillomavirus-initiated (HPV+) and HPV-negative (HPV−) disease, most HPV– cases arise from the oral cavity and larynx, with a smaller number of cases originating from the hypopharynx and oropharynx. Tobacco use and alcohol consumption are major risk factors for laryngeal carcinomas, which account for nearly 25% of SCCHN and are associated with high recurrence after treatment and limited overall survival (OS). Patients who are candidates for organ preservation are typically treated with a combination of chemotherapy and radiation, whereas those with high T stages often undergo surgery followed by adjuvant treatment.

Because treatments can be associated with significant morbidity, there is intense interest in identifying prognostic biomarkers and defining tumor subtypes that more accurately identify high-risk patients who may benefit from intensified therapy or novel targeted therapy trials. Molecular characterization of SCCHN to identify tumor subclasses associated with clinical outcomes^1, 2^, using mutation^3, 4^, epigenomic^5, 6^, and expression profiling^1, 7^, including The Cancer Genome Atlas (TCGA)^8^, has focused on tumors of the oral cavity and oropharynx, and the biology of HPV+ vs. HPV– disease^1, 2, 9, 10^. The TCGA analysis identified laryngeal cancer subclasses, of atypical, classic, basal, and mesenchymal types based on mRNA profiling. However, these subclasses were not prognostic. Other studies using TCGA data have shown prognostic differences in SCCHN subtypes, including larynx, based on differences in intratumor heterogeneity^10^. However, no comprehensive analyses have identified prognostic molecular differences informative for the mechanism.

In this study, we analyzed data for SCCHN using a recently developed integrative clustering approach^11^ that simultaneously assesses DNA mutation, copy number variation (CNV), methylation, and gene expression, for 256 cases of SCCHN for which complete data for these parameters and clinical prognosis are available in the TCGA. This analysis identified two distinct clusters of laryngeal cancer, associated with strong prognostic value, and showed that mutations in the genes *NSD1* and *NSD2* entirely segregated to the cluster with favorable prognosis. Subsequent validation in an independent set of 63 laryngeal cancer samples again demonstrated that mutations in *NSD1* or *NSD2* are independent favorable prognostic biomarkers for laryngeal cancer.

## Results

### Integrative clustering of SCCHN

**Fig. 1:**
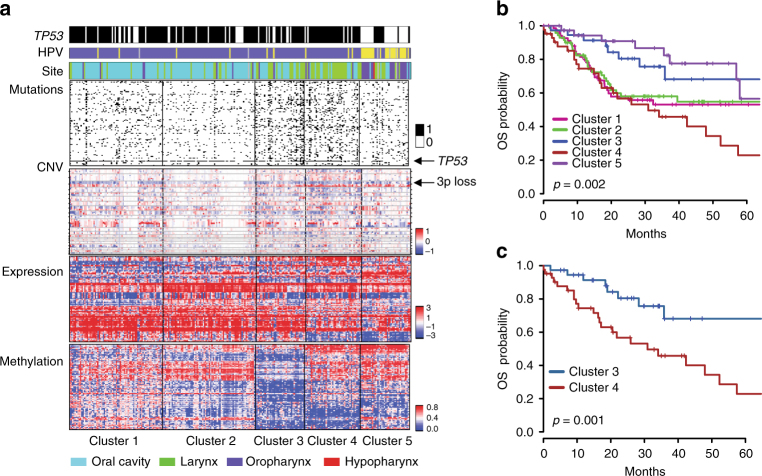
Integrative clustering of 256 SCCHN tumors. **a** The top three rows indicate *TP53* mutation (black), HPV status (purple = HPV–; yellow = HPV + ; and gray = unknown), and tumor site. Mutation panels indicate individual mutations in the top 25% most commonly mutated genes; note the marked mutational burden in clusters 3 and 4. CNV indicates the heatmap of chromosomal gains (red) and losses (blue). For gene expression, the heatmap indicates overexpression (red) or underexpression (blue) of genes; specific gene IDs are provided in Supplementary Data 1, and gene ontology (GO)-enriched categories are provided in Supplementary Data 2. For methylation, a heatmap plots β values of the probes as unmethylated (blue) or methylated (red). Genes corresponding to probes and enriched GO categories for methylation are listed in Supplementary Data 1 and 3, respectively. **b** Kaplan–Meier (KM) overall survival (OS) curves for all clusters identified in **a**. The *p-*value (log-rank test) indicates a strong evidence against the null hypothesis that the risk of death is similar across all SCCHN clusters. **c**. KM OS curves for clusters 3 and 4

Using the methods recently described by Mo et al.^11^, we performed integrative analysis for 256 cases of SCCHN for which complete data on DNA mutation, CNV, methylation, and gene expression were available in the TCGA. This distinguished 5 clusters that segregate tumors by disease site and HPV status (Fig. 1a; Supplementary Data 1, 2, and 3), and are associated with differences in OS (Fig. 1b). Overall, 73% (25/34) of HPV+ tumors, predominantly derived from the oropharynx, fell in cluster 5, marked by better prognosis (2-year OS 87%, 95% CI 68.8–95.0%), as has been previously reported for this disease type^8, 9^. The 152 HPV– oral cavity tumors were found predominantly in clusters 1 (39%) and 2 (37%), with the remaining 24% distributed throughout clusters 3, 4, and 5. While *TP53* mutations were predominant in oral and laryngeal clusters, mutation of *TP53* was low in cluster 5 (27%), reflecting the high incidence of HPV + oropharyngeal tumors, where *TP53* is inactivated posttranslationally by the virally encoded E6 oncoprotein^2^. The recently described prognostic 3pdel/TP53 subclass^12^ (Supplementary Fig. 1) is common in all HPV– clusters except cluster 2 (6%).

Most laryngeal tumors segregated to clusters 3 (25%) and 4 (39%), with the remaining 36% distributed evenly across the other clusters. SCCHN tumors in cluster 3 were associated with significantly higher OS vs. those in cluster 4 (2-year OS: 81.6% (95% CI 63.3–91.3%) vs. 54.6% (95% CI 37.9–68.5%); log-rank test: *p* = 0.001; Fig. 1c). This marked survival difference between laryngeal tumors in clusters 3 and 4 was intriguing, because the existence of two different laryngeal subtypes of prognostic value based on molecular profiles has not been previously described.

### Identification of prognostic laryngeal cancer clusters

**Fig. 2:**
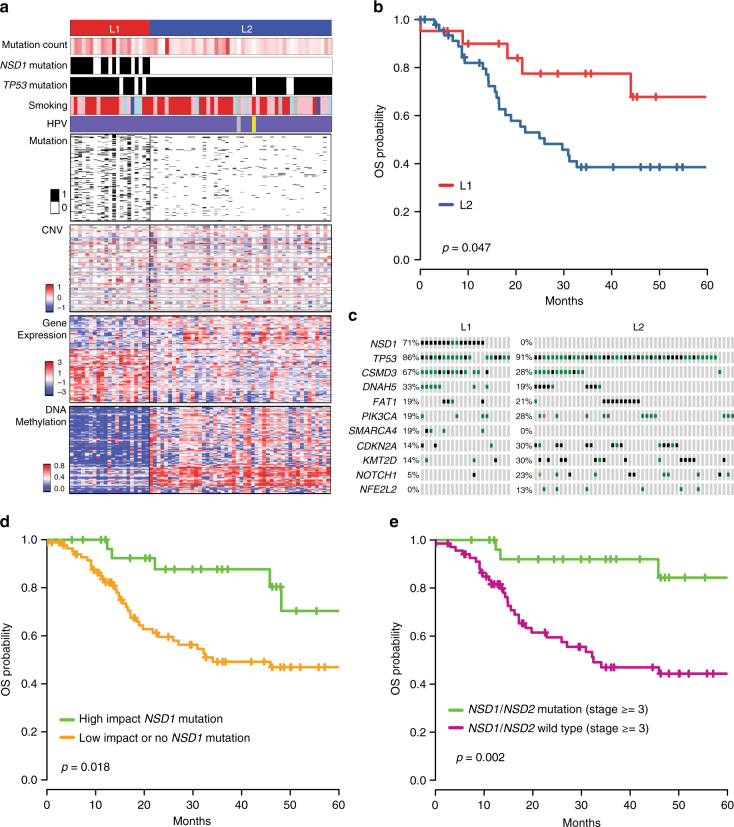
Integrative clustering of 69 laryngeal tumors. **a** The top panel shows larynx L1 (red) and L2 (blue) clusters. Mutation count for each sample is provided as a color gradient. *NSD1* and *TP53* bars indicate mutations in these genes (black). Smoking status (red = current smoker; pink = former smoker < 15 years; cyan = former smoker > 15 years; blue = lifelong nonsmoker; gray = status unavailable) and HPV status (purple = HPV–; yellow = HPV+; gray = unknown) are indicated. Also see Fig. 1 Legend and Methods. **b** Kaplan–Meier curves depicting OS of L1 and L2 clusters (*p*-value from log-rank test). **c** Mutation grid map of genes commonly mutated in L1 and L2. Each vertical bar represents a patient; black dot = truncating mutation; green = missense mutation. **d** KM curves for 116 laryngeal samples with NSD1 mutation impact determined by MutationAssessor^39^, Polyphen, and other function prediction algorithms. **e** KM curves for higher-stage laryngeal tumors (clinical stage 3 and 4) based on the presence or absence of predicted damaging mutations in *NSD1* or *NSD2*

Given that laryngeal tumors were distributed into two major clusters with differences in OS, we repeated integrative clustering analysis focusing solely on the 69 laryngeal tumors, resulting in two distinct clusters, L1 and L2 (Fig. 2a). These clusters had a significant difference in survival, with a 2-year recurrence-free survival (RFS) probability of 77.5% (95% CI 50.1–91%) vs. 44.7% (95% CI 29.6–58.7%; log-rank test: *p* = 0.010), and OS probability of 77.5% (95% CI 50.1–91%) vs. 53% (95% CI 37–66.7%; log-rank test: *p* = 0.047) (Fig. 2b and Supplementary Fig. 2a). No significant differences in age, gender, race, TNM stage, or reported smoking or alcohol status distinguished the clusters (Supplementary Data 4, 5, 6, and 7). Cluster L2, associated with poor outcome, differed from cluster L1 in methylation (Supplementary Data 8 and 9) and gene expression (Supplementary Data 8 and 10), as well as lower mutation burden (mean 169.4 vs. 258.9 per tumor; Wilcoxon rank sum test: *p* = 0.014), but not copy number changes. Among the genes reported as most commonly mutated in laryngeal cancer (including *TP53*, *CDKN2A*, *PIK3CA*, *DNAH5*, *NOTCH*, *FAT1*, *NFE2L2*, and *NSD1*), most of them were mutated at similar frequencies in L1 and L2 (Fig. 2c). However, *NSD1* was a striking exception, with inactivating mutations limited to L1 (Fisher’s exact test: *p* < 0.001) (Fig. 2a, c and Supplementary Fig. 3a), and 15 of 21 tumors displaying such mutations. Further analysis of inactivating *NSD1* mutations across the 5 SCCHN clusters indicated that these mutations concentrate in cluster 3 (Supplementary Data 4 and 5). Although *NSD1* mutations were detected in oral tumors (Supplementary Data 11), the association of *NSD1* mutation with better OS and RFS was specific to laryngeal tumors, suggesting a biological difference between tumor subsites (Supplementary Figs. 2b and 3b).

### Damaging mutations in *NSD1* and *NSD2* are prognostic of OS

**Fig. 3:**
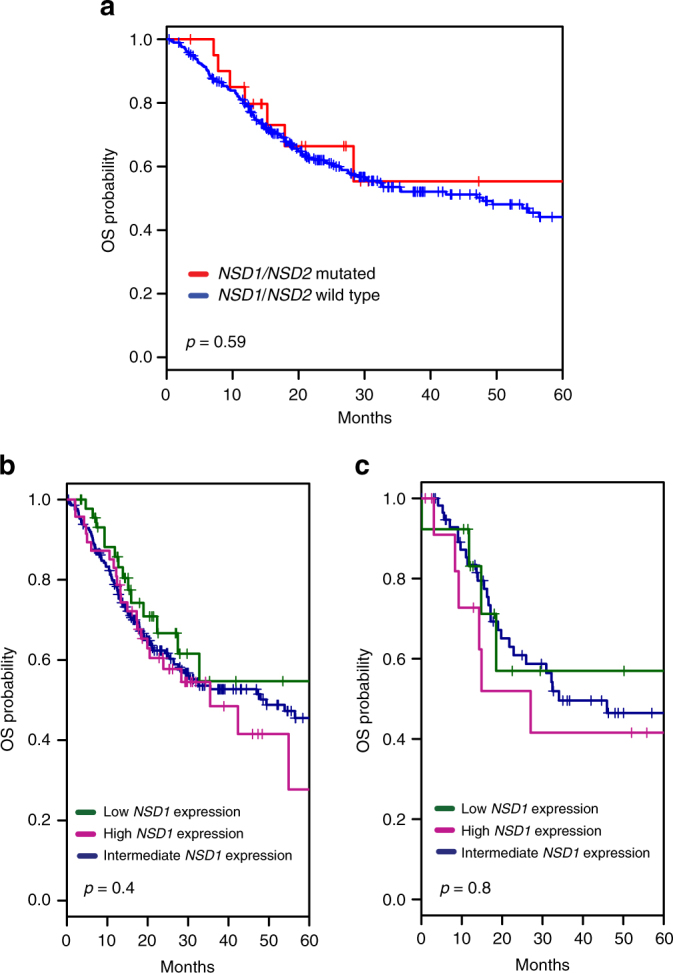
Kaplan–Meier curves for overall survival (OS) based on *NSD1/NSD2* mutation and *NSD1* expression status in nonlaryngeal and laryngeal tumors, based on TCGA data. **a** Kaplan–Meier curves showing OS for nonlaryngeal HPV– (including oral, oropharynx, and hypopharynx) SCCHN. Red and blue curves show *NSD1-* and *NSD2-* mutated, and *NSD1/NSD2* wild-type cases, respectively; OS differences are not significant (*p*-value from log-rank test). **b** Kaplan–Meier curves showing OS differences for patients with *NSD1* wild-type oral cancers trichotomized based on low (< or 15% quantile), intermediate (>15% and <85% quantiles), or high (>85% quantile) *NSD1* expression. **c** Kaplan–Meier curves showing OS for patients with *NSD1/NSD2* wild-type laryngeal cancers, with *NSD1* expression trichotomized as in **b**

We therefore analyzed the mutational profile of *NSD1* in the complete TCGA set of 116 laryngeal tumors for which at least mutational data were available among a total of 526 SCCHN cases (Supplementary Data 4 and 5). Among *NSD1* mutations in these tumors, 30 were predicted to be highly damaging based on multiple algorithms (Supplementary Data 5). In this expanded cohort, a significant association with improved OS (log-rank test: *p* = 0.018) was seen for high-impact *NSD1* mutations (2-year OS: 87.7%; 95% CI 66.3–95.9%) in comparison to low-impact or no *NSD1* mutations (2-year OS 59.6%; 95% CI 47.1–70%) (Fig. 2d). We further extended this analysis, restricting investigation of OS to stage 3 and 4 tumors to increase specimen homogeneity, and also including consideration of the less-frequently mutated *NSD1* paralog *NSD2*. This resulted in a striking increase in association between *NSD1/2* mutation and OS, with a median OS of 95 months for patients with either gene mutated (2-year OS: 92%; 95% CI 71.6–97.4%), vs. 32.5 months for those with both genes of wild type (2-year OS: 59.4%; 95% CI 45.6–70.8%; log-rank test: *p* = 0.002) (Fig. 2e). In contrast, similar analysis of highly damaging *NSD1* and *NSD2* mutations in HPV– oral, oropharynx, and hypopharynx tumors did not reveal a significant association with OS (Fig. 3a). No significant association was observed between *NSD1* or *NSD2* mutational status and tumor stage in larynx (Fisher’s exact test: *p* = 0.35; Table 1) and oral tumors (Fisher’s exact test: *p* = 0.3). The results were specific to mutation rather than altered gene expression, as among cases with *NSD1* and *NSD2* wild-type alleles in oropharynx and hypopharynx tumors (Fig. 3b) and laryngeal tumors (Fig. 3c), *NSD1* mRNA expression levels did not predict survival. Altogether, these observations that indicated loss-of-function mutations in *NSD1* or *NSD2* are associated with better prognosis specifically in laryngeal cancer.

**Table 1:** Results from statistical analyses for association between clinical characteristics and NSD1 and NSD2 (NSD) mutation status for laryngeal samples in the discovery (TCGA) and validation cohorts

		TCGA					Validation				
		NSD1/2 mutated, *n* = 32		Wild type, *n* = 84		*p*-value*	NSD1/2 mutated, *n* = 24		Wild type, *n* = 39		*p*-value*
		*n*	%	*n*	%		*n*	%	n	%	
*Patient Characteristics*											
Age, yrs Median (range)		61.5 (38–80)		62 (39–83)		0.51	51.5 (36–86)		65 (44–83)		<0.001
Age, yrs group						0.49					<0.001
	36–49	4	12.5	7	8.3		11	45.8	2	5.1	
	50–69	24	75.0	59	70.2		10	41.7	25	64.1	
	70–86	4	12.5	18	21.4		3	12.5	12	30.8	
Gender						1.00					0.022
	Female	5	15.6	15	17.9		9	37.5	4	10.3	
	Male	27	84.4	69	82.1		15	62.5	35	89.7	
Race						0.15					0.88
	Black	2	6.3	17	20.2		5	20.8	7	17.9	
	White	29	90.6	62	73.8		18	75.0	31	79.5	
	Other/unknown	1	3.1	5	6.0		1	4.2	1	2.6	
Smoking status						0.34					
	Current	19	59.4	39	46.4		smoking data unavailable				
	Former	13	40.6	36	42.9						
	Never	0	0.0	6	7.2						
	Unknown	0	0.0	3	3.6						
Alcohol use						0.75	alcohol data unavailable				
	Yes	23	71.9	53	63.1						
	No	9	28.3	29	34.5						
	Unknown	0	0.0	2	2.4						
*Tumor Characteristics*											
Clinical stage						0.35					0.72
	Stage I,II	4	12.5	12	14.3		5	20.8	12	30.8	
	Stage III	11	34.4	16	19.1		7	29.2	7	18.0	
	Stage IV	16	5.0	53	63.1		10	41.7	17	43.6	
	Stage unknown	1	3.1	3	3.6		2	8.3	3	7.7	
Node status						0.67					0.58
	Node Neg (N0)	18	58.1	39	49.4		12	54.5	23	63.9	
	Any positive (N+)	13	41.9	40	50.6		10	45.5	13	36.1	
	Unknown^a^	1		5			2		3		

^*^ *p*-value is from Fisher’s exact test for categorical variables or Wilcoxon rank- sum test for age (continuous)

^a^Nodal status “unknown” excluded from comparison by mutation status

**Fig. 4:**
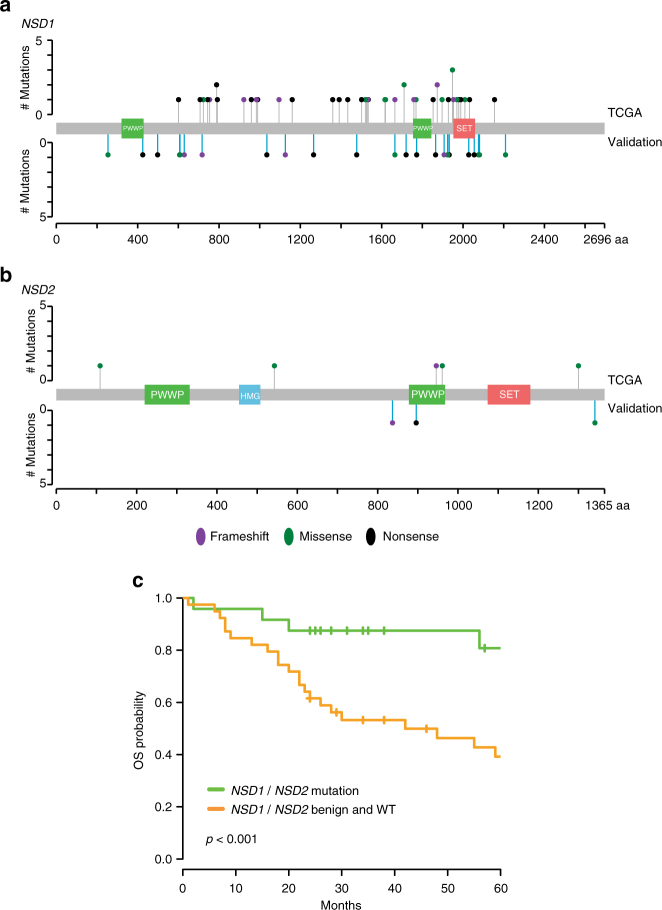
Validation of *NSD1* and *NSD2* mutations in an independent laryngeal cancer cohort. **a** Plot indicates the location of nonsense (black), frameshift (purple), and missense (green) mutations affecting the NSD1 protein. NSD1 functional domains are indicated. Lollipops above the line indicate mutations reported in the TCGA. Lollipops below the line depict mutations identified in the validation cohort. **b** Plot similar to **a**, for NSD2. **c** Kaplan–Meier curve indicates OS of patients with laryngeal tumors with damaging *NSD1* or *NSD*2 mutation (*n* = 24) or without such mutations (*n* = 39), in the validation cohort (*p*-value from log-rank test)

To validate these results, we compiled a set of 63 previously unstudied laryngeal tumors from surgical resections obtained before any treatment, including chemotherapy or irradiation (Table 1). Using Fluidigm amplification and Illumina sequencing, we identified damaging mutations for *NSD1* (21/63) (Fig. 4a) or *NSD2* (3/63) (Fig. 4b) in 24 of the 63 cases, with nonsense (38%) and missense (29%) mutations predominating. In addition to these, we identified nine patients with gene variants predicted to be nondamaging, with several known to be nonrare variants based on data in the Exome Aggregation Consortium (ExAC)^13^: we considered these as benign (Supplementary Data 12). The 24 patients with damaging *NSD1* or *NSD2* mutations had extended OS (2-year OS: 87.5%; 95% CI: 66.1–95.8%) vs. the 39 with benign or no mutations (2-year OS: 61.5%; 95% CI: 44.5–74.7%) (log-rank test: *p* < 0.001) (Fig. 4c). In extended comparison of the 116 TCGA specimens and of the 63 validation specimens with defined NSD1/NSD2 mutation status (Table 1), no significant differences in race, nodal status, or clinical stage distinguished patients with laryngeal tumors with or without these mutations. No significant association was found with age and gender in the TCGA cohort; interestingly, in the validation cohort, which contained a number of patients diagnosed at an earlier age, NSD1/2 mutation was associated with earlier diagnosis (Wilcoxon rank sum test: *p* < 0.001) and gender (Fisher’s exact test: *p* = 0.022).

**Fig. 5:**
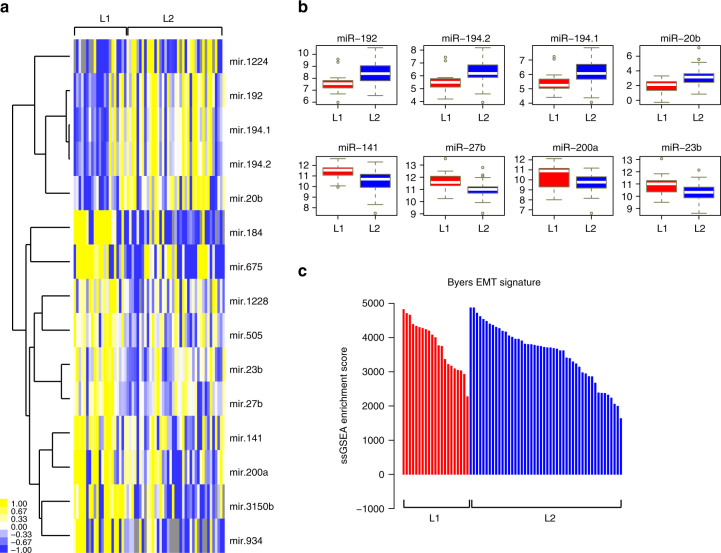
Enrichment profiles of EMT signatures and microRNAs in L1 and L2. **a** Heatmap showing miRNAs with significant differences in expression profile between the L1 and L2 clusters. Blue indicates underexpression, yellow indicates overexpression, and gray indicates expression data not available. **b** Box plot showing the normalized gene counts (reads per million miRNA mapped) of miRNAs that are over- or underexpressed in L1 clusters of laryngeal tumors. *X*-axis indicates the L1 and L2 clusters, while the *Y*-axis indicates normalized gene counts of miRNA for both L1 (blue box) and L2 (red box) samples. **c** Waterfall plot depicting the enrichment score for each laryngeal tumor against a gene set of 76 genes forming an EMT signature as defined by Byers et al.^15^, using the ssGSEA algorithm. Blue and red bars indicate tumors in L1 and L2 clusters, respectively. *Y*-axis shows the enrichment scores. A positive enrichment score indicates the extent to which genes in EMT signature are overexpressed in each tumor sample. Similarly, a negative enrichment score indicates the extent to which genes in EMT signature are downregulated in the tumor sample

One study has suggested a relationship between *NSD1* depletion, DNA hypomethylation, and differential expression of some miRNAs^8^, including enriched expression of miR-200a/b, which is involved in epithelial–mesenchymal transition (EMT)^14^. However, that work analyzed a combined set of oral and laryngeal tumors. Considering only the laryngeal tumor set, we find several miRNAs to be differentially expressed between L1 and L2 (Fig. 5a). However, no selective enrichment for miRNAs associated with EMT distinguished these clusters. Further, enrichment analysis for an EMT^15^ mRNA signature showed comparable enrichment in L1 and L2 (Fig. 5b, c), likely reflecting the advanced stage of most laryngeal specimens in the TCGA. These results bear further investigation in an extended laryngeal cohort for which mRNA and miRNA data are available, since as an important caveat, the lack of significant association might, in part, represent the limited statistical power available when considering only laryngeal cases. However, extending our analysis, we carried out Gene Set Enrichment Analysis (GSEA)^16^ comparing genes differentially expressed between L1 and L2 (differing by 775 genes with DESeq2 Wald test^17^: *p* < 0.001; Supplementary Data 13). Interestingly, this identified several gene sets (Supplementary Fig. 4 and Supplementary Data 14) involved in stem cell maintenance and hypoxic response that are elevated in L2, compatible with the poorer overall survival of patients in this class.

## Discussion

Management of laryngeal cancer is complex where therapeutic resistance contributes to significant morbidity. Moreover, in patients that are candidates for total laryngectomy have decreased quality of life. Therefore, prognostic biomarkers for therapeutic intensity and predictive biomarkers for radiosensitivity (and thus suitability for larynx preservation) are urgently needed. Using integrative clustering of four different genomic data of SCCHN including mutation, copy number, gene expression, and methylation obtained from TCGA, we identified a novel laryngeal cluster with a significant difference in OS where the prognostic laryngeal cancer subtype is associated with mutations in *NSD1* and *NSD2*.

**Fig. 6:**
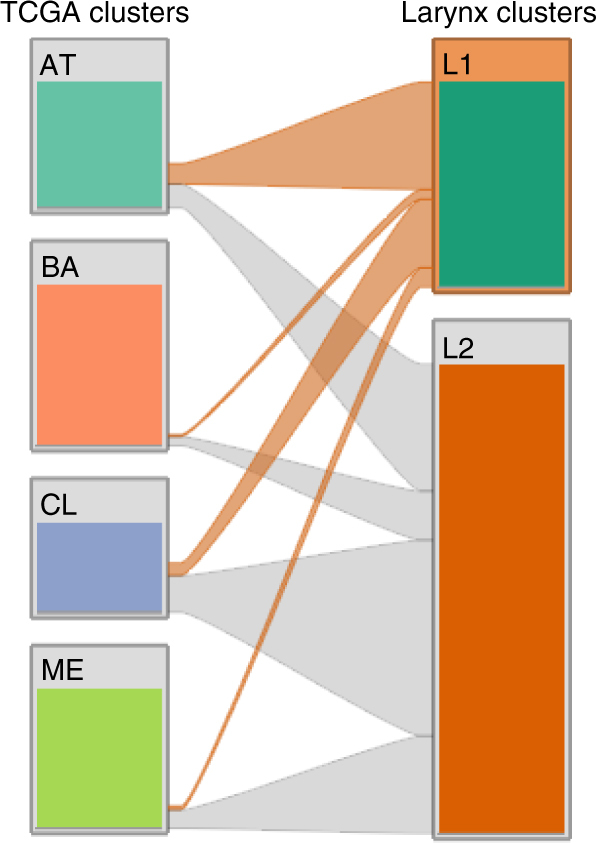
Relationship between SCCHN TCGA (mRNA derived) subtypes, and integrative clustering-derived laryngeal subtypes assigned in this study. The left column represents the distribution of laryngeal tumors among the 256 SCCHN specimens (laryngeal, oral, oropharynx, and hypopharynx) assigned by the TCGA as atypical (AT), basal (BA), classical (CL), and mesenchymal (ME) subtypes. Gray and orange bands indicate the redistribution of TCGA laryngeal tumors to the L1 and L2 prognostic clusters defined in this work (right). The plot shows that the majority of tumor samples in L1 cluster belong to the atypical (52%) and classical (33%) subtypes, whereas the majority of L2 tumor samples belong to classical (42%), atypical (27%), and mesenchymal (21%) subtypes

Inherited mutations in *NSD1* cause Sotos syndrome^18^, and are implicated in a number of cancers^19^, as is mutation of *NSD2* ^20^. As lysine methyltransferases, *NSD1* and *NSD2* influence gene expression by methylating specific histone sites (H3K36, H4K20) and nonhistone targets such as *NF-κB* ^19, 21, 22^. Mutations in *NSD1* have been associated with extensive demethylation of DNA in CpG islands at promoters, as well as intergenic regions^8, 23^, compatible with the significantly lower levels of methylation observed in cluster L1 (Fig. 2a, Supplementary Data 15 and 16). Using germline DNA from Sotos syndrome patients, it has been shown that loss of *NSD1* function is associated with extensive DNA hypomethylation^24^. H3K36 methylation is known to induce localized activity of DNA methyltransferases^25–27^; for example, the de novo DNA methyltransferase DNMT3B is recruited to gene body regions that are enriched for H3K36me3 by SETD2^25, 28^. Interestingly, a recent publication that showed decreased dimethylation of lysine in H3 histones (H3K36me2) in cases of SCCHN was associated with damaging *NSD1* mutations^29^, although this study did not identify a prognostic role for *NSD1* mutations, consider a possible role for *NSD2*, or assign prognostic value specifically to tumors of the larynx. While they have been reported as tumor suppressive in neuroblastoma and glioma^30^, amplification and overexpression of *NSD1*  have been reported as oncogenic in some settings, suggesting context-dependent action^31^. While other studies^1, 3, 4, 8^ have cataloged distinct prognostic molecular features for HPV + and oral tumors, this work for the first time distinguishes molecular subclasses of laryngeal tumors with markedly different prognosis, with a positive outcome specifically associated with mutation of *NSD1* and *NSD2*. This classification markedly differs from earlier TCGA analyses, which segregated laryngeal tumors into non-prognostic “classical” and “atypical” subclasses lacking the separation of *NSD1* ^8^ (Fig. 6; Table 2). Developmentally, the larynx differs significantly from the oral cavity, with the larynx, trachea, and lung arising from the endoderm and a common lung bud, while the oral cavity and hypopharynx arise from a complex interplay of ectodermal and endodermal lineages^32^. Such lineage differences may underlie distinct activities of *NSD1* and *NSD2* in these SCCHN settings.

**Table 2:** Relationship between SCCHN TCGA (mRNA derived) subtypes, and integrative clustering-derived laryngeal subtypes assigned in this study

TCGA clusters	L1 larynx cluster	L2 larynx cluster
Atypical (AT)	11 (52%)	13 (27%)
Basal (BA)	1 (5%)	5 (10%)
Classical (CL)	7 (33%)	20 (42%)
Mesenchymal (ME)	2 (10%)	10 (21%)

Numbers of samples and percentage of intersection between laryngeal clusters and TCGA clusters

The fact that mutations associated with hypomethylation of histones and DNA is provocative for laryngeal cancer, given the emergence of DNA demethylating agents such as azacitidine (5-aza-CR) and decitabine (5-aza-CdR) as promising agents in clinical trials^33^. Our data suggest that demethylating agents and EZH2 inhibitors may be particularly effective in tumors lacking *NSD1* or *NSD2* mutations, and clinical trials seeking to establish survival benefits for these drugs in patients with laryngeal tumors should restrict study populations to those with *NSD* wild-type tumors. Also of potential clinical relevance, the methyltransferase EZH2 selectively methylates H3K27, and several clinical trials are evaluating the efficacy of EZH2 inhibition in treating solid tumors and hematologic malignancies^34, 35^. Although the use of EZH2 inhibitors in the context of *NSD* mutations has not been studied, one recent report suggested a promising mechanism for application of EZH2 inhibitors in *NSD2*-overexpressing myeloma cells^36^.

Our study suggests that *NSD* mutation status may play an important role in the efficacy of such inhibitors in patients with laryngeal cancer. Finally, although no significant association between clinical parameters (i.e., tumor stage and nodal status) and NSD1/2 mutation status was observed in either the discovery or validation sets, further evaluation in additional independent cohorts is required. In sum, this work nominates *NSD1* and *NSD2* as rapidly exploitable prognostic biomarkers and targets for functional scrutiny in laryngeal cancers.

## Methods

### Clustering of molecular data for all SCCHN cases

Molecular data sets describing somatic mutations, CNV, gene expression based on RNA-seq, and methylation for 256 SCCHN cases were obtained from the TCGA data portal (https://tcga-data.nci.nih.gov/tcga/) (August 2014 data freeze) and The Cancer Genome Atlas Genome Data Analysis Center (GDAC) Firehose website (https://confluence.broadinstitute.org/display/GDAC/). To maintain coherency of analysis across different data layers and cancer types, we used normalized data classified as levels 2 and 3 in the TCGA data hierarchy^37^ as the starting point of our analysis. Of the 528 samples that are available from TCGA consortium, 256 samples had profiling data for all 4 genomic data sets (mutations, CNV, gene expression, and methylation), and were used for integrative clustering.

For somatic mutation data, all genes with nonsynonymous or nonsense variants from among these 256 cases were identified and used to construct a binary matrix after applying a 5% mutation frequency cutoff to remove infrequently mutated genes. For copy number variation data, we applied the strategy described in the supplement of Mo et al.^11^ to obtain a set of nonredundant regions and representative copy number quantifications for each region. Germline copy number variants and regions from chromosomes X and Y were removed from the data using standard approaches^38^. For detailed analysis to identify *NSD1* missense variants as deleterious or benign, we used the MutationAssessor/Functional Impact Score (FIS)^39^, Polyphen, and a Grantham score available through SeattleSeq annotation server (http://snp.gs.washington.edu/SeattleSeqAnnotation138/). MutationMapper^40^ was used to display mutations graphically. For analysis of OS associated with *NSD1* mutation in larynx cancer, the full set of 116 laryngeal tumors present in the 526-specimen SCCHN TCGA collection containing mutational data was analyzed.

For gene expression data, gene-level RPKM values (reads per kilobase per million mapped reads) from the TCGA data portal were log_2_-transformed and subjected to a variation filter: the 5000 genes with the highest median absolute deviation (MAD) across all 256 SCCHN specimens were retained. Similarly, level 3 methylation data (β values from the Illumina HumanMethylation450k bead chip) were filtered to retain 2000 probes with the highest MAD across samples. These β values were then logit-transformed into M-values^41^.

The resulting four matrices were used as input to integrative clustering analysis using iClusterPlus^11^. A plot relating the percentage of explained variation to the number of clusters suggested five clusters as an appropriate choice for clustering the 256 SCCHN samples (Supplementary Fig. 5a). To analyze segregation of 3p loss and *TP53* mutation among the clusters, a median of the segmentation mean (MSM) values between chromosome 3p25 and 3p13 was calculated. Cases with MSMs < −0.3^42^ are identified as cases with 3p loss. These samples were further segregated into two classes based on the mutant and wild-type status of *TP53* gene.

### Clustering of data to define L1 and L2 classes

We also applied the methods described above to cluster the 69 laryngeal samples among the 256 fully characterized SCCHN samples. The processing of the data described above for expression, CNV, and methylation was applied to larynx samples. A submatrix of the SCCHN mutation matrix representing the larynx samples was extracted. The resulting data matrices were analyzed using iClusterPlus; given the relatively small sample size, a two-cluster solution was selected (Supplementary Fig. 5b). The resulting data were depicted as a heatmap showing mutation, copy number, gene expression, and methylation. The mutation counts were shown as a gradient where red and white indicate maximal (1463) and minimal mutation counts (3) (Fig. 2a). The mutational burden between L1 and L2 clusters was compared by applying Wilcoxon rank sum test.

In order to identify methylation differences distinguishing the L1 and L2 laryngeal clusters, we applied the following data-processing steps. First, probes with all β values less than 0.1 were removed. Next, probes were mapped to 158,848 CpG islands and 6127 promoter sites, and were aggregated to obtain median M-values for each such region. These steps were performed in R using the Bioconductor package wateRmelon^43^. Subsequently, we applied Wilcoxon rank sum tests to compare methylation levels between L1 and L2. FDR-adjusted *p*-values were calculated with the Benjamini–Hochberg method^44^, and a 5% FDR cutoff was applied to select differentially methylated regions.

We identified differentially expressed genes between clusters L1 and L2 using DESeq2^17^, which models RNA-seq read counts with a negative binomial distribution. A total of 775 genes were identified as differentially expressed (Wald test *p* < 0.001 and log2-fold change > 1 and < −1) and were used for enrichment analysis with Gene Set Enrichment Analysis (GSEA)^16^, which was run using default parameters and ‘classic’ as the enrichment statistic. For *NSD1* wild-type SCCHN tumors, to evaluate whether NSD1 gene expression was associated with survival differences in laryngeal or oral cancer cases, we used Voom-normalized^45^ RSEM (RNA-Seq by Expectation Maximization) expression data. *NSD1* expression was trichotomized into low (<or = 15% quantile), intermediate (>15% and <85% quantile), or high (> or = 85% quantile), and survival distributions were estimated and compared using Kaplan–Meier method and log-rank test, respectively, on the resulting groups.

We evaluated the differential miRNA expression between L1 and L2 clusters based on miRNA quantification reported as reads per million miRNA mapped, establishing significance by applying Wilcoxon rank sum tests. FDR-adjusted *p-*values were calculated with Benjamini–Hochberg method, and a 5% FDR cutoff was applied to select differentially expressed miRNAs. To visualize the differentially expressed miRNAs, we applied hierarchical clustering on miRNA expression data using average linkage clustering with Pearson’s uncentered correlation as a distance measure. The resulting clustered data table was used as input to Java TreeView (http://jtreeview.sourceforge.net) to generate a heatmap. In order to assess the enrichment of epithelial–mesenchymal transition signatures in L1 and L2 clusters, we applied single-sample GSEA (ssGSEA)^46^ using the EMT signatures curated from Byers et al.^15^ and the available gene sets from MSigdb resource. The resulting enrichment scores were depicted as waterfall plots.

The functional significance of genes that contributed to segregation of five SCCHN and two laryngeal clusters was assessed using enrichment of gene ontology terms (GO) (biological process and molecular function) present in the DAVID bioinformatics resources^47^ (Supplementary Data 2, 3, 8, and 9). To identify and depict the relationship between tumors from L1 and L2 clusters and gene expression- derived subtypes defined by TCGA, we obtained 279 tumor class labels indicating the subtypes from TCGA^8^. We stratified the relationship between these two groups using StratomeX tool^48^. In a StratomeX plot (Fig. 6),  columns indicate TCGA mRNA classification (atypical, basal, classical, and mesenchymal) and laryngeal L1 and L2 tumors. The bands between these two groups indicate the intersection of tumor samples between two groups. The width of the band shows the level of intersection.

### Validation of *NSD1/2* mutations and prognostic significance

A validation set for laryngeal tumors was compiled from the biosample registries of Sidney Kimmel Cancer Center at Johns Hopkins School of Medicine (JH) and Fox Chase Cancer Center (FCCC) (with clinical characteristics summarized in Table 1). Study protocols from both institutions were reviewed and approved by the institutional review board (IRBs) from both institutions, and informed consent was received from all human patients included in the study. All tumors from JH cohort were fresh frozen tumors, while FCCC samples were formalin-fixed paraffin embedded (FFPE). All patients who were treated with chemotherapy or radiotherapy prior to their surgery were excluded, as were tumor samples from patients with synchronous diseases including esophageal and lung cancers. We included those cases for which definitive dates for diagnosis, treatment modalities, disease recurrence or metastasis, and time of death or last contact were available. A total of 99 samples were identified from both JH (*n* = 68) and FCCC (*n* = 31) biosample repositories for evaluation of NSD1/2 mutation status. Based on exclusion criteria, we excluded 15 and 7 samples from JH and FCCC cohort from the analysis, respectively. Samples with low DNA yield or quality were eliminated (JH: *n* = 3; FCCC: *n* = 11). After accounting for exclusion and inclusion criteria listed above, we obtained 63 (JH: *n* = 50; FCCC: *n* = 13) samples from both institutions. All samples were reviewed by a pathologist to reconfirm the diagnosis prior to DNA extraction. Frozen tissues were cut into 5-μm sections, stained with H&E, and examined by light microscopy. Lesions with a low neoplastic cellularity (<70%) were additionally microdissected to remove contaminating normal cells before DNA extraction. Paraffin-embedded slides were microdissected to obtain > 70% neoplastic cells. Neoplastic cellularity was estimated from the sequential slides, which highly reflect the cellularity of the section used for DNA sequencing.

### DNA isolation

Genomic DNA was isolated from fresh frozen samples by the QIAamp DNA Kit (Qiagen). DNA extractions from FFPE-derived material were performed using standard protocols as previously described^49^. All samples were quantified using a DeNovix NanoDrop spectrophotometer (Wilmington, DE).

### *NSD1*/*NSD2* amplification

Primers pairs (91 for NSD1 and 49 for NSD2) were designed by Fluidigm (San Francisco, USA) to cover all exons of the NSD1/NSD2 genes and exon–intron boundaries. A total of 100 ng of genomic DNA from neoplastic lesions were used for PCR amplification of target genes using an Access Array™ microfluidic chip according to the manufacturer’s instructions. Each sample was combined with primer pairs in a microfluidic chip, and thermal cycling on a Fluidigm FC1 Cycler was performed. PCR products were then collected using the integrated fluidic circuit controller and transferred to a 96-well plate. In a separate PCR, Illumina sequence-specific adaptors and barcodes were attached. Prior to sequencing, all amplified samples were checked on the Agilent 2100 BioAnalyzer to determine if the PCR product in the DNA reaction has the expected size. All fresh frozen samples passed the quality control (QC). Twelve FFPE-derived samples did not pass the QC and were excluded from sequencing. A total of 50 fresh frozen and 13 FFPE cases were considered for subsequent downstream analysis.

### Sequencing

Pooled and indexed PCR products were sequenced on the Illumina MiSeq instrument following standard protocols with the following modifications: Illumina-specific sequencing primers were substituted with a mixture of two Fluidigm-specific primer pairs (FL1 and FL2). Quality and coverage metrics for MiSeq sequencing data indicated a mean coverage depth of 2000 reads. The sequencing reads were aligned to human genome (Hg19) using BWA^50^. The resulting BAM files were processed using the GATK best-practices pipeline^51^ with no mark duplicate step. We applied the Mutect2 algorithm (based on Mutect^52^) with default parameters using the tumor samples-only mode. Variants were filtered at an 8% variant allele fraction^53^. The resulting VCF files were processed using ANNOVAR^54^ for functional annotation of identified variants, and with independent variant calling using HaplotypeCaller (GATK) and VarScan^55^ for comparison. Most of the NSD1/2 damaging variants were identified by all three variant callers. In assessing the functional impact of missense variants, we applied a rule that at least 3 functional impact prediction algorithms (viz. SIFT^56^, Polyphen2^57^, LRT^58^, Mutation Assessor^39^, and FATHMM^59^) predict functionally damaging variants. For predicting overall survival differences among *NSD1/2* loss-of-function mutations, we combined benign- and wild-type alleles into one group.

### Survival analyses

Clinical information including follow-up and new tumor event (NTE) data for 526 SCCHN samples (including the 256 samples chosen for clustering) were obtained from the TCGA website (July 2015). OS and RFS distributions were estimated using Kaplan–Meier methods. OS was based on vital status and “days to death” (CDE_ID:3165475) from initial pathologic diagnosis. Individuals who were still alive at the time of the last follow-up were censored. The RFS outcome included time to first recurrence or death (with an individual censored if neither event was observed prior to the last follow-up). Recurrence was defined as the appearance of either “locoregional recurrence” or “distant metastasis”, and time to first recurrence was estimated from “days to new tumor event after initial treatment” (CDE_ID: 3392464). As this item starts at the end of initial treatment rather than the date of pathologic diagnosis, the recurrence-free interval may be underestimated; data on the interval from diagnosis to initial treatment were not available in this data freeze. Survival curves were compared with log-rank tests, and these calculations were done using the R ‘survival’ package^60^. To test the association between clinical parameters (and the presence of mutations) and cluster membership, Fisher’s exact or Wilcoxon rank sum tests were used. These tests were two-sided with 5% type 1 error. Wherever necessary, significance levels of multiple comparisons were corrected for type 1 error rates using Benjamini–Hochberg false-discovery rate (FDR) method^44^. To test the association between clinical parameters (and the presence of mutations) and cluster membership, or the presence/absence of damaging mutations of *NSD1* or *NSD2* genes, two-sided Fisher’s exact and Wilcoxon rank sum tests were used, and *p*-values < 0.05 were considered significant.

### Data availability

De-identified patient variant data that support the findings of this study are available from the corresponding authors upon request, and are submitted to the database of Genotypes and Phenotypes (dbGaP). Primary results from data analyses were done using standard R and Bioconductor packages including iClusterPlus and Survival. R scripts calling routines from these packages will be available from the corresponding author upon request.

## Electronic supplementary material

Supplementary Information

Description of Additional Supplementary Files

Supplementary Data 1

Supplementary Data 2

Supplementary Data 3

Supplementary Data 4

Supplementary Data 5

Supplementary Data 6

Supplementary Data 7

Supplementary Data 8

Supplementary Data 9

Supplementary Data 10

Supplementary Data 11

Supplementary Data 12

Supplementary Data 13

Supplementary Data 14

Supplementary Data 15

Supplementary Data 16
