# Supplementary material for: NSD1- and NSD2-damaging mutations define a subset of laryngeal tumors with favorable prognosis
Source: Nat Commun. 2017 Nov 24;8:1772. doi: 10.1038/s41467-017-01877-7 (PMC5701248; doi:10.1038/s41467-017-01877-7)
Supplement: Supplementary file 1 — Supplementary Information [file 41467_2017_1877_MOESM1_ESM.pdf]

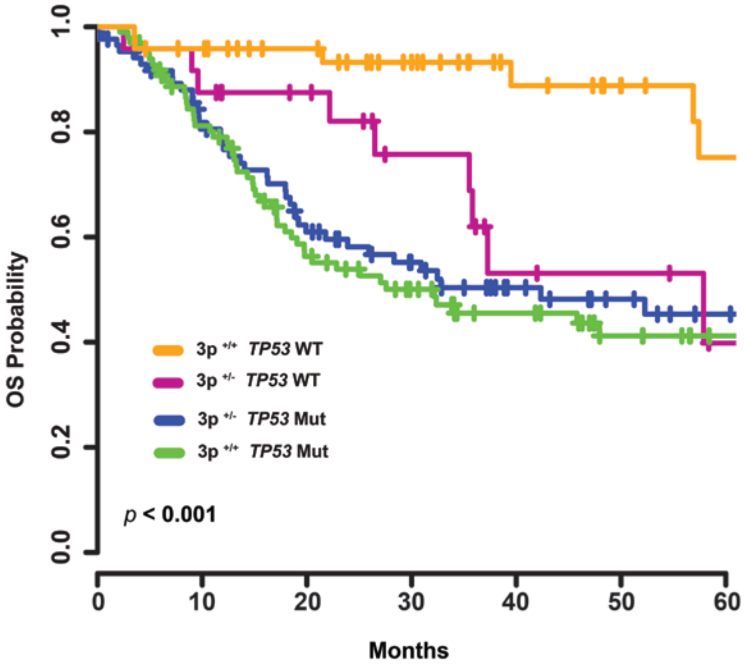

Supplementary Figure 1. **Analysis of distribution of HNSCC cases with 3p loss and *TP53* mutation among the 5 defined clusters, from 256 HNSCC cases.** Kaplan-Meier analysis indicates marked differences in overall survival (OS) of cases involving four different combinations of *TP53* mutation and 3p loss shown in Supplementary Table 1. The *p*-value is calculated using log-rank test.

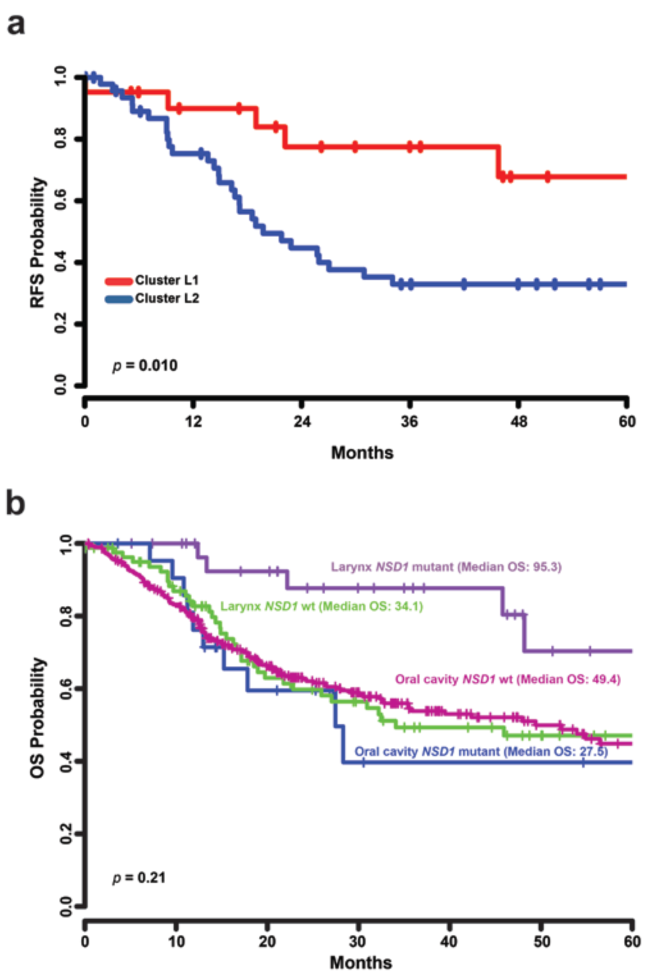

Supplementary Figure 2. **Kaplan-Meier curves showing relapse free survival and overall survival.** **a**. Kaplan-Meier curves showing the probability of recurrence-free survival (RFS) of cases in the L1 and L2 classes defined in Figure 2a. See text for details. **b**. Kaplan-Meier curves showing overall survival for cases with and without *NSD1* mutation among larynx and oral tumor samples. Numbers in parenthesis indicate estimated median overall survival.

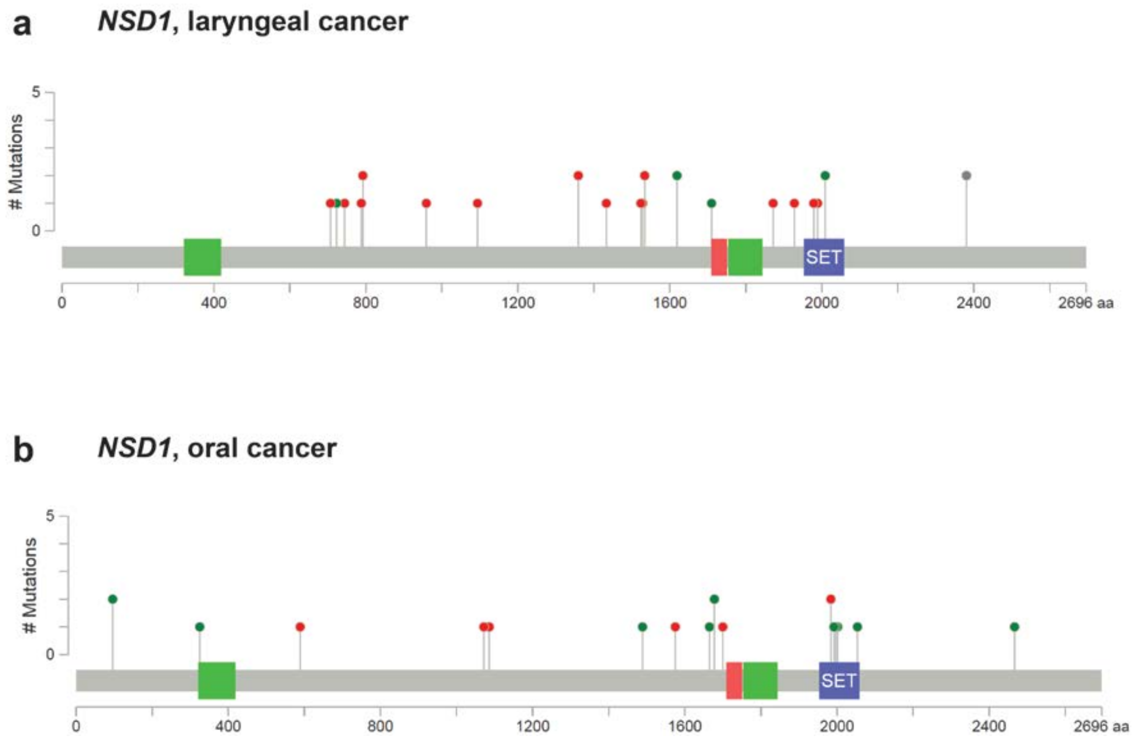

Supplementary Figure 3. Map of *NSD1*-targeting mutations. **a**. MutationMapper plot in which vertical lollipops indicate location of nonsense/frameshift (red), missense (green) and silent (gray) mutations affecting the *NSD1* protein in 15 laryngeal cancers from the L1 cluster. **b.** MutationMapper plot for 14 oral cancer samples with *NSD1* mutations, as in **a**.

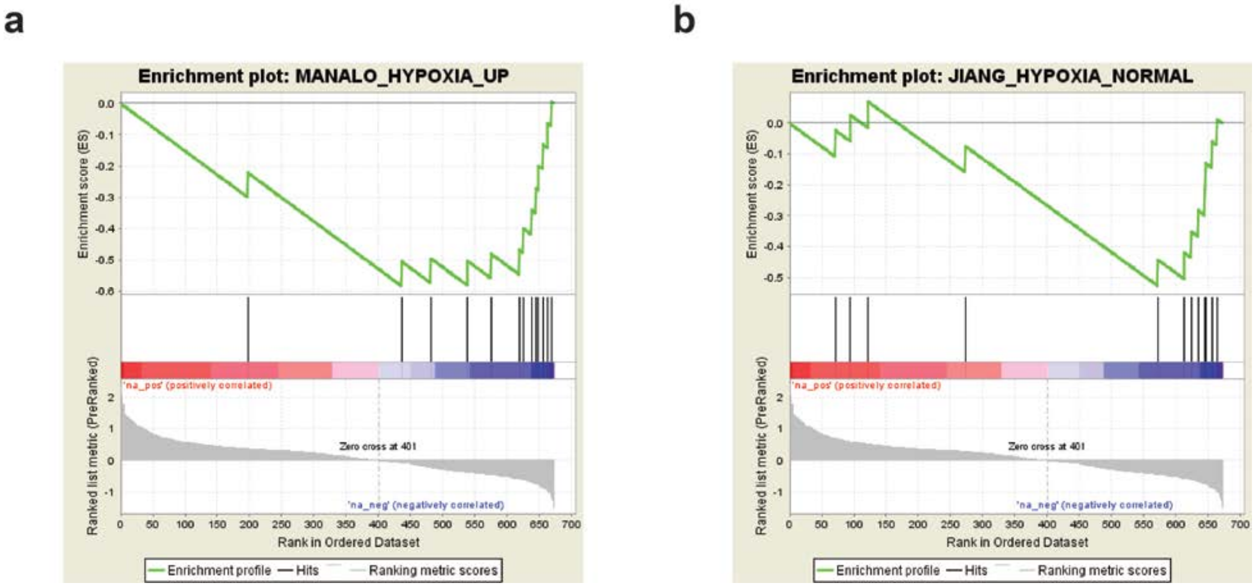

Supplementary Figure 4. Enrichment plots for hypoxia gene sets. Genes that are differentially (based on DESeq2) expressed between L1 and L2 (L1/L2) were used for GSEA analysis. **a**. Genes overexpressed in the L2 cluster are enriched for those up-regulated in response to both hypoxia and overexpression of an active form of *HIF1A* in arterial endothelial cells. **b**.Genes overexpressed genes in L2 are enriched for those up-regulated by hypoxia in normal renal proximal tubule epithelial cells. X axis indicates the rank order for all genes (fold difference over-expressed to under-expressed in L1). Y axis (green line) indicates the running enrichment score for the gene set. Presence of a differentially expressed gene in a gene set results in deviation of green curve as the analysis walks along the ranked list. The score at the peak of the plot is the enrichment score (ES) for this gene set and those genes appear before or at the peak are defined as core enrichment genes for this gene set. Red bar indicates genes over-expressed in L1 and blue bar indicate genes under-expressed in L1 or over-expressed in L2. Deviation of green line away from 0 and peaking at -0.5 or -0.6 indicate negative-enrichment of genes in the given gene set in L1. The central portion of the plot indicates where members of gene set appear on the ranked list. The bottom panel (gray scale) tracks the value of the ranking metric across the list of ranked genes, indicating genotype/phenotype correlation. A positive value indicates correlation with the L1 phenotype and a negative value indicates correlation with the L2 phenotype.

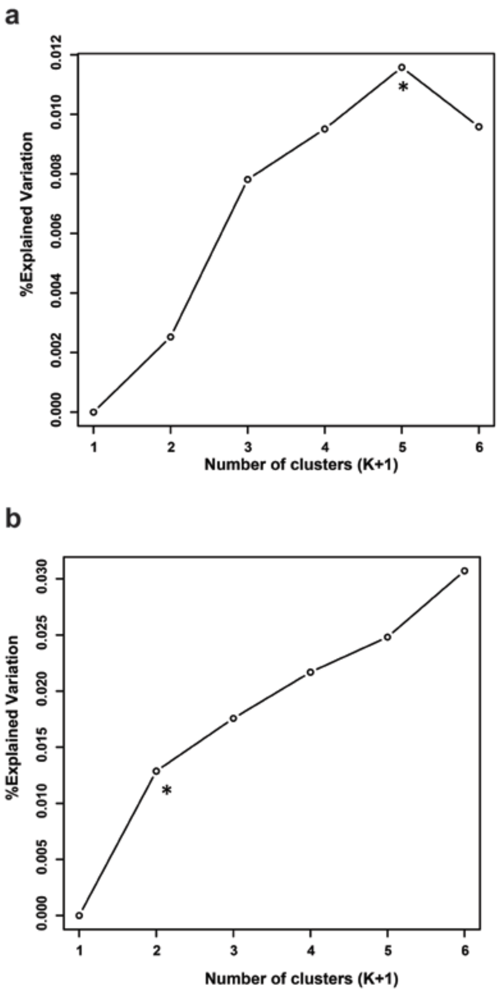

Supplementary Figure 5. Model selection. Plots showing percent of explained variation vs. number of clusters. The optimal number of clusters for integrative clustering can be selected where this curve flattens out and beyond which increases in percent of explained variation are minimal. **a**. In the classification of 256 SCCHN tumors, a 5-cluster solution provides a meaningful variation. **b**. In the case of Larynx (69 samples), a 2-cluster solution was chosen to retain a meaningful sample size within each cluster.

	Clusters				
	**1**	**2**	**3**	**4**	**5**
3p loss + *TP53* mutation	34	4	21	23	4
3p loss + *TP53* WT	6	0	3	2	13
3p intact + *TP53* mutation	21	51	7	13	6
3p intact + *TP53* WT	9	15	6	4	14
Total	70	70	37	42	37

Supplementary Table 1: Table showing distribution of 3p loss and *TP53* mutation among 5 defined clusters from 256 HNSCC samples (from Figure 1). The values indicate the number of samples that satisfy the criterion in each category, with the total number of specimens in each cluster indicated.
