# Supplementary material for: NSD1- and NSD2-damaging mutations define a subset of laryngeal tumors with favorable prognosis
Source: Nat Commun. 2017 Nov 24;8:1772. doi: 10.1038/s41467-017-01877-7 (PMC5701248; doi:10.1038/s41467-017-01877-7)
Supplement: Supplementary file 2 — Description of Additional Supplementary Files [file 41467_2017_1877_MOESM2_ESM.pdf]

Description of Additional Supplementary Files

File Name: Supplementary Data 1
Description: List of genes and probes that are differentially expressed and methylated between clusters 1-5, respectively (Fig 1a).

File Name: Supplementary Data 2
Description: List of enriched gene ontology (GO) terms (biological processes and molecular functions; modified Fisher's exact test: $p < 0.01$) for genes where gene expression differences contributed to clustering of 5 SCCHN clusters.

File Name: Supplementary Data 3
Description: List of enriched gene ontology (GO) terms for biological processes and molecular functions (modified Fisher's exact test: $p < 0.01$) for genes where DNA methylation differences contributed to clustering of 5 SCCHN clusters.

File Name: Supplementary Data 4
Description: Data showing the demographic information for SCCHN samples obtained from TCGA data repository. Data show mutational status for NSD1 and NSD2 in the samples, samples that were considered for clustering analysis with cluster designations. Data also shows those samples that were not considered for clustering analyses.

File Name: Supplementary Data 5
Description: List of 116 laryngeal samples with and without *NSD1* mutations obtained from TCGA and GADC Firehose MAF files. Damaging impact of these mutations were calculated using MutAssessor, Polyphen, and Grantham scores available through the SeattleSeq annotation server (hg19). The Data also indicates the copy number status and methylation status of *NSD1* and p16 (*CDKN2A*).

File Name: Supplementary Data 6
Description: Tumor node status and *NSD1* mutation status for laryngeal samples in L1 and L2 clusters.

File Name: Supplementary Data 7
Description: List of 34 laryngeal tumor cases for which cisplatin treatment information is available from TCGA clinical data Datas.

File Name: Supplementary Data 8
Description: List of genes, where gene expression and DNA methylation differences contributed to clustering of L1 and L2 laryngeal cluster.

File Name: Supplementary Data 9
Description: List of enriched gene ontology terms (biological processes and molecular functions; modified Fisher's exact test: $p < 0.01$) for genes where DNA methylation differences contributed to clustering of two laryngeal clusters L1 and L2.

File Name: Supplementary Data 10
Description: List of enriched gene ontology terms (biological processes and molecular functions; modified Fisher's exact test: $p < 0.01$) for genes where gene expression differences contributed to clustering of two laryngeal clusters L1 and L2.

File Name: Supplementary Data 11
Description: List of 32 SCCHN samples with *NSD1* mutations in non-laryngeal sites (oral, hypopharynx and oropharynx).

File Name: Supplementary Data 12
Description: List of *NSD1* and *NSD2* mutations identified in the validation cohort.

File Name: Supplementary Data 13
Description: List of differentially expressed genes between larynx L1 and L2 clusters calculated using DeSeq2 algorithm.

File Name: Supplementary Data 14
Description: List of GSEA enriched gene sets (at 25% FDR) for genes differentially expressed between larynx L1 and L2 clusters. A – shows list of gene sets enriched for genes overexpressed in Larynx L2 cluster. B – Shows list of gene sets enriched for genes underexpressed in L2, C – List of genes that contributed to positive enrichment of hypoxia gene sets in L2.

File Name: Supplementary Data 15
Description: List of differentially methylated regions located in the gene regulatory regions (promoters) between larynx L1 and L2 clusters.

File Name: Supplementary Data 16
Description: List of differentially methylated regions located in the CpG islands (CGI) between larynx L1 and L2 clusters.
